# A Potential circRNA-miRNA-mRNA Regulatory Network in Asthmatic Airway Epithelial Cells Identified by Integrated Analysis of Microarray Datasets

**DOI:** 10.3389/fmolb.2021.703307

**Published:** 2021-07-16

**Authors:** Dian Chen, Wenliang Wu, Lingling Yi, Yuchen Feng, Chenli Chang, Shengchong Chen, Jiali Gao, Gongqi Chen, Guohua Zhen

**Affiliations:** ^1^ Division of Respiratory and Critical Care Medicine, Department of Internal Medicine, Tongji Hospital, Tongji Medical College, Huazhong University of Science and Technology, Wuhan, China; ^2^ Key Laboratory of Respiratory Diseases, National Health Commission of People’s Republic of China, and National Clinical Research Center for Respiratory Diseases, Wuhan, China

**Keywords:** asthma, airway epithelial cells, microarray datasets, mRNA, microRNA, circular RNA, robust rank aggregation, integrated analysis

## Abstract

**Background:** Asthma is one of the most prevalent chronic respiratory diseases worldwide. Bronchial epithelial cells play a critical role in the pathogenesis of asthma. Circular RNAs (circRNAs) act as microRNA (miRNA) sponges to regulate downstream gene expression. However, the role of epithelial circRNAs in asthma remains to be investigated. This study aims to explore the potential circRNA-miRNA-messenger RNA (mRNA) regulatory network in asthma by integrated analysis of publicly available microarray datasets.

**Methods:** Five mRNA microarray datasets derived from bronchial brushing samples from asthma patients and control subjects were downloaded from the Gene Expression Omnibus (GEO) database. The robust rank aggregation (RRA) method was used to identify robust differentially expressed genes (DEGs) in bronchial epithelial cells between asthma patients and controls. Gene Ontology (GO) and Kyoto Encyclopedia of Genes and Genomes (KEGG) enrichment analyses were used to annotate the functions of the DEGs. Protein-protein interaction (PPI) analysis was performed to identify hub genes. Three miRNA databases (Targetscan, miRDB, and miRWalk) were used to predict the miRNAs which potentially target the hub genes. A miRNA microarray dataset derived from bronchial brushings was used to validate the miRNA-mRNA relationships. Finally, a circRNA-miRNA-mRNA network was constructed via the ENCORI database.

**Results:** A total of 127 robust DEGs in bronchial epithelial cells between steroid-naïve asthma patients (*n* = 272) and healthy controls (*n* = 165) were identified from five mRNA microarray datasets. Enrichment analyses showed that DEGs were mainly enriched in several biological processes related to asthma, including humoral immune response, salivary secretion, and IL-17 signaling pathway. Nineteen hub genes were identified and were used to construct a potential epithelial circRNA-miRNA-mRNA network. The top 10 competing endogenous RNAs were *hsa_circ_0001585*, *hsa_circ_0078031*, *hsa_circ_0000552*, *hsa-miR-30a-3p*, *hsa-miR-30d-3p*, *KIT*, *CD69*, *ADRA2A*, *BPIFA1*, and *GGH*.

**Conclusion:** Our study reveals a potential role for epithelial circRNA-miRNA-mRNA network in the pathogenesis of asthma.

## Introduction

Asthma is a heterogeneous disease and is characterized by chronic airway inflammation. Over 300 million people are suffering from asthma worldwide nowadays and the number is expected to reach more than 400 million by the year 2025 (To et al., 2012; Barcik et al., 2020; Global Strategy for Asthma Management and Prevention, 2020). Approximately 250, 000 confirmed deaths are reported annually worldwide due to respiratory failure during asthma exacerbations (Christiansen and Zuraw, 2019). Airway epithelial cells play a pivotal role in asthma pathogenesis, including airway inflammation, mucus overproduction, airway wall remodeling, and bronchial hyperresponsiveness (Gohy et al., 2020; Hellings and Steelant, 2020; Hammad and Lambrecht, 2021). However, the intrinsic molecular mechanisms of epithelial cells in asthma are still not fully clarified.

Circular RNAs (circRNAs), which comprise a large proportion of stable RNAs in eukaryotes, have been identified in large quantities owing to the widespread use of high-throughput RNA sequencing and the development of bioinformatics-based algorithms (Jeck et al., 2013; Memczak et al., 2013; Salzman et al., 2013; Wang et al., 2014; Gao and Zhao, 2018; Chen, 2020). CircRNAs are produced by the so-called backsplicing mechanism, a process in which a downstream 5′ donor site is covalently linked to an upstream 3′ acceptor site to form a stable closing RNA structure containing exon and/or intron sequences (Jeck et al., 2013; Memczak et al., 2013; Salzman et al., 2013; Kristensen et al., 2019; Chen, 2020). Notwithstanding a lack of 5′ and 3′ ends through the non-canonical splicing, circRNAs are generally thought to localize to the cytoplasm. Therefore, circRNAs might function as microRNA (miRNA) sponges and sequester miRNA away from mRNAs, thus indirectly regulate gene expression (Hsu and Coca-Prados, 1979; Jeck et al., 2013; Memczak et al., 2013; Chen, 2020). Such competing endogenous RNAs (ceRNA) mechanism has been considered a prominent function of circRNAs. Previous studies have revealed that circRNAs are involved in multiple biological processes including neuronal function (Piwecka et al., 2017; Kleaveland et al., 2018), pluripotency regulation (Yu et al., 2017), cell differentiation (Kristensen et al., 2018), and cancer progression (Wang et al., 2019a; Wong et al., 2020) via decoying miRNAs. MiRNAs constitute another major class of non-coding RNAs with approximately 22 nucleotides (Gebert and MacRae, 2019; Goodall and Wickramasinghe, 2021). Previous reports have demonstrated that miRNAs can bind mRNAs through partial complementarity and reduce gene expression by restraining mRNA translation and/or facilitating mRNA degradation (Yekta et al., 2004; Lewis et al., 2005; Gebert and MacRae, 2019; Goodall and Wickramasinghe, 2021). However, the role of circRNA-miRNA-mRNA networks in airway epithelial cells in asthma pathogenesis has not been reported yet.

Currently, data mining from Gene Expression Omnibus (GEO) database has been applied in asthma studies. However, the studies based on a single dataset with a limited sample size will lead to deviation and bias in data interpretation. Integrating multiple datasets to include more samples was a promising strategy to increase the credibility of the analysis. Using the robust rank aggregation (RRA) method can reduce the heterogeneity of different experiments with different analysis platforms.

In this study, we integrated five mRNA microarray datasets from the GEO database utilizing the RRA method to identify the robust DEGs in bronchial epithelial cells between asthma patients and healthy controls, based on which a potential circRNA-miRNA-mRNA regulatory network was constructed. Our study reveals a potential role for epithelial circRNA-miRNA-mRNA network in the pathogenesis of asthma.

## Material and Methods

### Microarray Datasets Acquisition and Processing

The mRNA and miRNA expression profiles of asthma patients were obtained from the GEO database (https://www.ncbi.nlm.nih.gov/geo/) utilizing the getGEO function of the GEOquery package (version 2.58.0) in R software (version 4.0.3) (Barrett et al., 2012). The following terms were used to search the microarray studies: “Asthma”, AND “epithelial cells”, OR “bronchial brushings”, AND “mRNA expression”, OR “miRNA expression”, AND “Homo sapiens”, AND “Microarray”. The following eligibility criteria were used to include or exclude datasets and samples: 1) A minimum of 10 subjects in the dataset, containing at least four asthma patients and four controls; 2) Bronchial brushings were used for microarray analysis; 3) All subjects were older than 18 years old and were steroid-naïve; 4) Raw data were available in GEO.

The expression matrix file and related platform annotation document of each microarray dataset were downloaded from the GEO database to convert the names of microarray probes to the gene symbols. Probes matching with multiple gene symbols were eliminated and the mean values were calculated for gene symbols corresponding to multiple probes. After excluding samples with strong heterogeneity according to the hclust method, the differentially expressed genes (DEGs) and the differentially expressed miRNAs (DEMis) between asthma and control samples in each dataset were identified by using the “limma” (linear models for microarray data) package (version 3.46.0) (Ritchie et al., 2015) in R software with the cut-off criteria of |log2 fold change| > 0.5 and *p*-value < 0.05.

### Robust Rank Aggregation Analysis

To integrate the outcomes of multiple microarray datasets and minimize the bias and inconsistencies, the RRA method was adopted to identify the robust DEGs. The up- and down-regulated DEGs were firstly ranked by expression fold changes in each dataset and were subsequently analyzed using the “Robust Rank Aggregation” R package (version 1.1) (Kolde et al., 2012). The score in the RRA result indicated the ranking degree of each gene in the final gene list. Genes with score < 0.05 and |log2 fold change| > 0.5 were considered as the significant robust DEGs.

### Visualization of Chromosome Locations of Robust Differentially Expressed Genes

The “RCircos” R package (1.2.1) was utilized to visualize the expression patterns and the chromosomal positions of all robust DEGs.

### Functional and Pathway Enrichment Analyses

Gene Ontology (GO) and Kyoto Encyclopedia of Genes and Genomes (KEGG) enrichment analyses were used to investigate the biological process (BP), the cellular component (CC), the molecular function (MF), and the involved pathways of selected molecules, which were performed with the “clusterprofiler” R package (version 3.18.1) (Yu et al., 2012). The GO terms and KEGG pathways with *p*-value < 0.05 were considered statistically significant and further visualized via the “ggplot2” R package (version 3.3.3). The KEGG enrichment analysis of differentially expressed miRNAs was conducted using the miRPathDB v2.0 (https://mpd.bioinf.uni-sb.de/) database (Kehl et al., 2020).

### DisGeNET Database Analysis

DisGeNET (http://www.disgenet.org) database is one of the largest available platforms of human disease-associated genes and variants through manually integrating the scientific literature (Piñero et al., 2017). For a given gene list, DisGeNET database can identify significantly correlated diseases.

### Protein-Protein Interaction Network Construction and Clusters Analysis

All previously identified robust DEGs were uploaded to the STRING (version 11.0) database (https://www.string-db.org/) to construct the protein-protein interaction (PPI) network (Szklarczyk et al., 2021). Confidence > 0.4 was set as the screening criteria. The PPI network was subsequently reconstructed and visualized via the Cytoscape (version 3.8.2) (http://cytoscape.org/) software (Su et al., 2014). In the Cytoscape plot, each node represented a gene/protein/miRNA/circRNA, while the edge between nodes represented the interactions of molecules. The molecular complex detection (MCODE) plugin of the Cytoscape software was used to screen out significant clusters in the PPI network.

### Hub Gene Identification

CytoHubba is another plugin of the Cytoscape software for ranking nodes in a network, which provides eleven topological analysis methods and six centralities to identify hub genes based on shortest paths, including Maximal Clique Centrality (MCC), Density of Maximum Neighborhood Component (DMNC), Maximum Neighborhood Component (MNC), Degree, Edge Percolated Component (EPC), BottleNeck, EcCentricity, Closeness, Radiality, and Betweenness (Chin et al., 2014). Considering the potential heterogeneity of the biological network, the RRA method was adopted to integrate the results of different analysis methods for catching essential proteins.

### GeneMANIA Database Analysis

GeneMANIA (http://www.genemania.org) database was used to construct the PPI network and explore the putative functions of up-loaded genes (Warde-Farley et al., 2010). For a given query list, GeneMANIA analyzes target genes with functionally similar genes together to obtain regulatory networks.

### CircRNA-miRNA-mRNA Network Construction

The Targetscan (http://www.targetscan.org/vert_72/) (Garcia et al., 2011), miRDB (http://mirdb.org/) (Chen and Wang, 2020), and miRWalk (http://mirwalk.umm.uni-heidelberg.de/) (Dweep et al., 2011) databases were used to predict the corresponding miRNAs of all 19 hub genes. The overlapping results of three databases were then intersected with the differentially expressed miRNAs of GSE142237. The ENCORI (http://starbase.sysu.edu.cn/index.php) database (Li et al., 2014) was used to predict the upstream circRNAs of the selected miRNA-mRNA pairs. The final ceRNA network was further processed with the Cytoscape software (version 3.8.2).

### Statistical Analysis

The differential analysis was conducted by the “limma” package (version 3.46.0) in R version 4.0.3. Heatmap was used to reveal the logarithmic fold changes of robust DEGs in the RRA analysis. *p* < 0.05 was considered statistically significant.

## Results

### Subjects Characteristics of the Microarray Datasets Included in This Study

Five mRNA microarray datasets (GSE4302, GSE43696, GSE63142, GSE67472, and GSE41861) and one miRNA microarray dataset (GSE142237) derived from bronchial epithelial brushings were obtained from the GEO database. There were a total of 272 steroid-naïve asthma patients and 165 healthy controls in the five mRNA microarray datasets. The miRNA microarray dataset (GSE142237) included a total of eight asthma patients and four healthy controls. Only asthma patients without any steroid treatments were included for further analysis.

**FIGURE 1:**
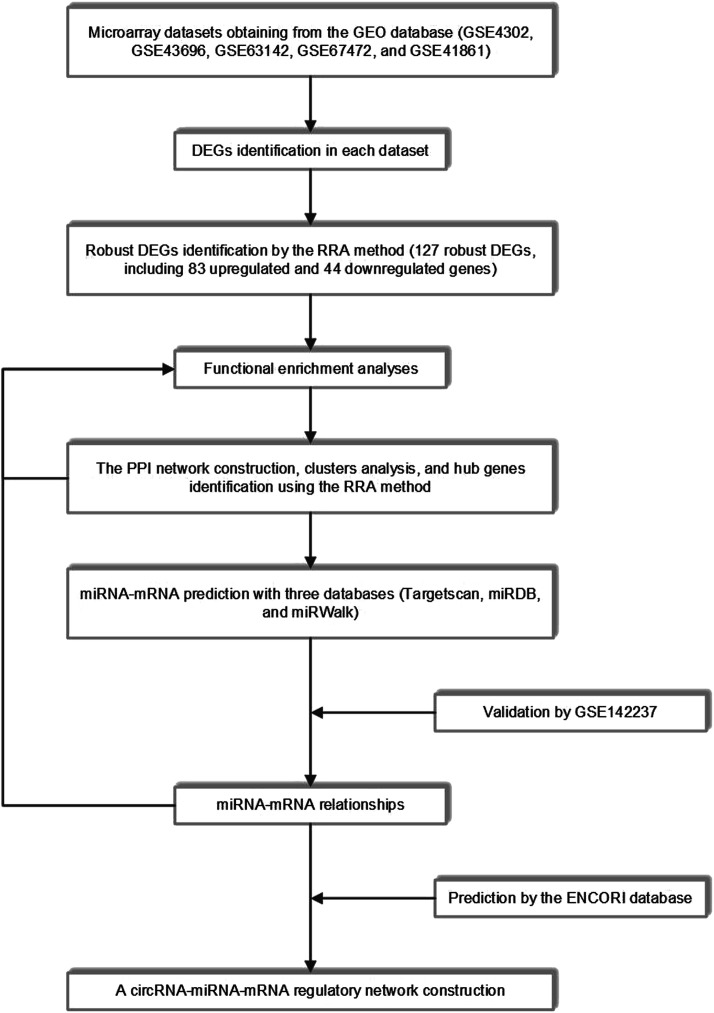
The whole study workflow. GEO, Gene Expression Omnibus; DEGs, differentially expressed genes; RRA, robust rank aggregation; PPI, protein-protein interaction.

The workflow of the study was shown in Figure 1. Detailed information on the datasets mentioned above was shown in Table 1.

**TABLE 1:** Characteristics of six microarray datasets included in the study.

GSE accession number	Participants	Data type	Samples	Platform	R Package	Year
GSE4302	74 asthma patients (42 steroid-naïve) and 28 healthy controls	mRNA	Bronchial brushings	GPL570	Limma	2007
GSE43696	88 asthma patients (50 steroid-naïve) and 20 healthy controls	mRNA	Bronchial brushings	GPL6480	Limma	2014
GSE63142	128 asthma patients (72 steroid-naïve) and 27 healthy controls	mRNA	Bronchial brushings	GPL6480	Limma	2014
GSE67472	62 asthma patients (steroid-naïve) and 43 healthy controls	mRNA	Bronchial brushings	GPL16311	Limma	2015
GSE41861	51 asthma patients (46 steroid-naïve) and 47 healthy controls	mRNA	Bronchial brushings	GPL570	Limma	2015
GSE142237	8 asthma patients (steroid-naïve) and 4 healthy controls	miRNA	Bronchial brushings	GPL18058	Limma	2019

### Identification of Differentially Expressed Genes in Steroid-Naïve Asthma Patients

**FIGURE 2:**
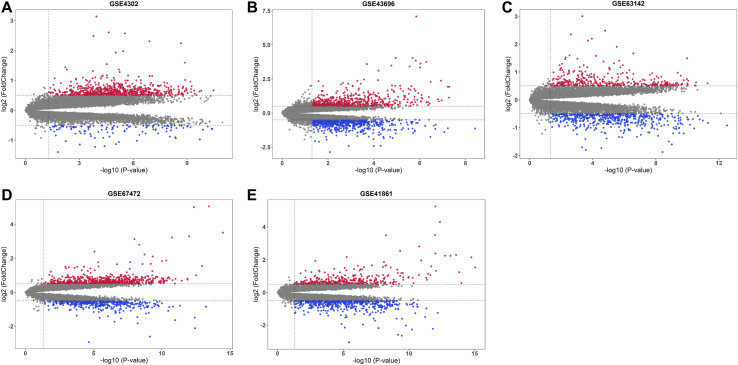
Volcano plots of five mRNA microarray datasets. The upregulated genes were marked in red, while the downregulated genes were marked in blue. The gray dots represented genes with no significant difference. **(A)** GSE4302; **(B)** GSE43696; **(C)** GSE63142; **(D)** GSE67472; **(E)** GSE41861.

After base two logarithm conversions, all five mRNA microarray datasets were further analyzed using the “limma” package of the R software to identify DEGs in primary airway epithelial cells between steroid-naïve asthma patients and healthy controls. The cut-off criteria were as follows: *p*-value < 0.05 and |log2 fold change| > 0.5. The distribution of DEGs in five datasets was illustrated by the volcano plots in Figure 2. There were 851 DEGs (737 upregulated and 114 downregulated), 1218 DEGs (606 upregulated and 612 downregulated), 783 DEGs (345 upregulated and 438 downregulated), 1084 DEGs (650 upregulated and 434 downregulated), and 875 DEGs (348 upregulated and 527 downregulated) in GSE4302, GSE43696, GSE63142, GSE67472, and GSE41861, respectively.

**FIGURE 3:**
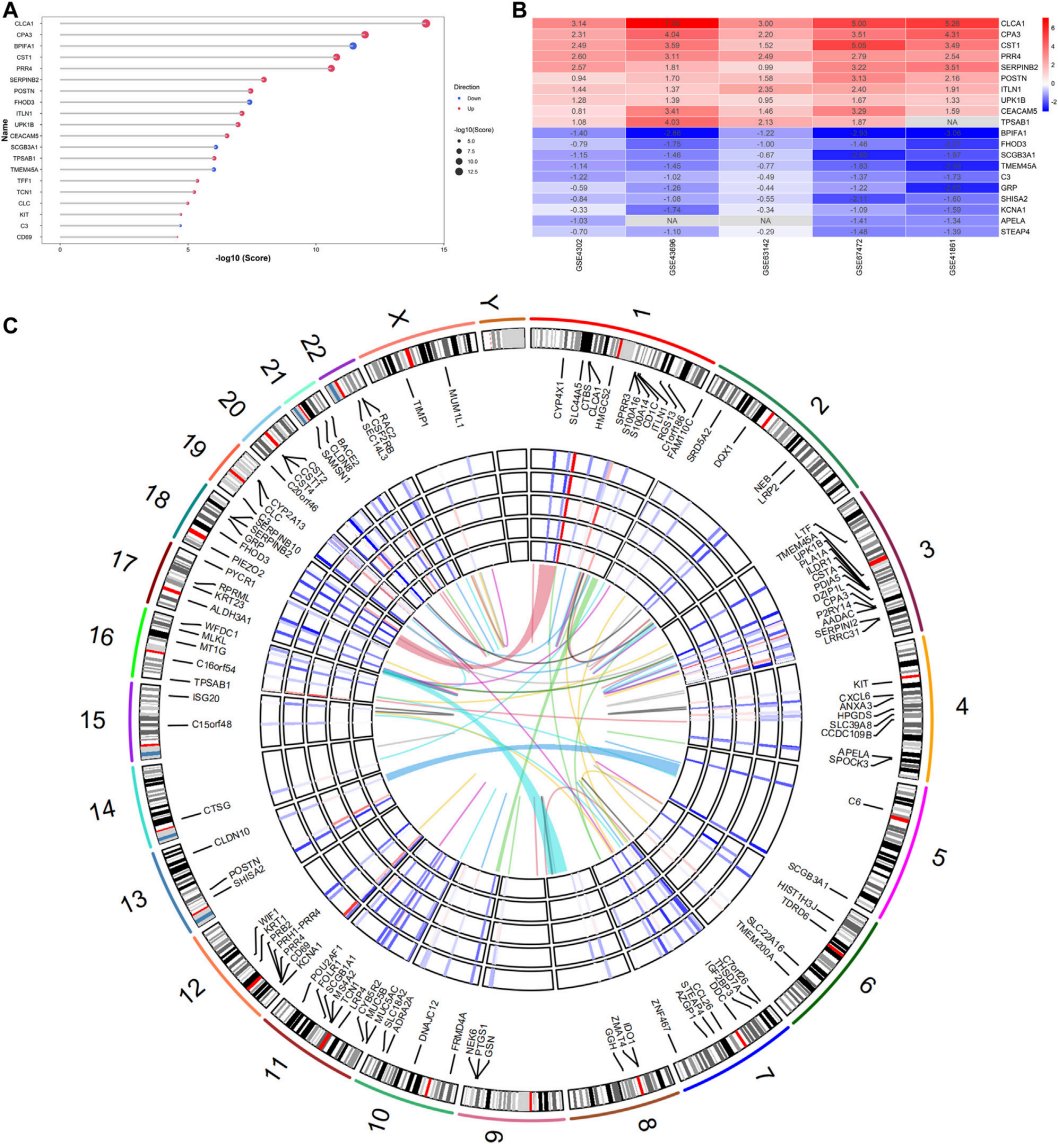
Identification of robust DEGs in the RRA analysis. **(A)** The lollipop chart showed the top 20 ranked genes identified by the RRA method. The red and blue dots represented the up- and down-regulated genes, respectively. Bigger dots represented higher ranks; **(B)** The heatmap showed the top 10 up- and down-regulated DEGs in five GEO series. Red represented high expression of DEGs in asthma patients, while blue represented low expression of DEGs in asthma patients. The numbers in the box indicated logarithmic fold changes in each dataset; **(C)** The circular heatmaps showed the chromosomal positions of all robust DEGs. The outer circle represented chromosomes, while the inner circle heatmaps represented logarithmic fold changes of all robust DEGs in five asthma microarray datasets.

The RRA analysis was conducted to integrate five mRNA microarray datasets with minimal bias and a total of 127 integrated DEGs were identified, including 83 upregulated and 44 downregulated robust DEGs. Lower RRA scores in the results indicated higher gene ranks and the top 20 genes were shown in the lollipop chart (Figure 3A), only five of which were downregulated. The top 10 ranked genes in asthma were composed of eight upregulated genes [including *CLCA1* (score = 4.99E-15), *CPA3* (score = 1.21E-12), *CST1* (score = 1.56E-11), *PRR4* (score = 2.51E-11), *SERPINB2* (score = 1.08E-08), *POSTN* (score = 3.57E-08), *ITLN1* (score = 7.85E-08), *UPK1B* (score = 1.11E-07)] and two downregulated genes [including *BPIFA1* (score = 3.52E-12), *FHOD3* (score = 3.90E-08)]. Of note, *CLCA1*, *SERPINB2*, and *POSTN*, as the IL-13-responsive genes and airway epithelial gene signatures for type 2 status, were all at a higher rank in five microarray datasets. This suggested the significant roles of the three genes in asthma pathogenesis and the reliability of the RRA results (Woodruff et al., 2007; Woodruff et al., 2009). The top 10 up- and down-regulated DEGs were drawn on the heatmap (Figure 3B). The expression patterns and the chromosomal positions of all robust DEGs across the five microarray datasets were shown in Figure 3C. These DEGs were distributed in all chromosomes except chromosome Y. Chromosome three contained the most DEGs.

### Functional Annotation of Robust Differentially Expressed Genes in Asthma

**FIGURE 4:**
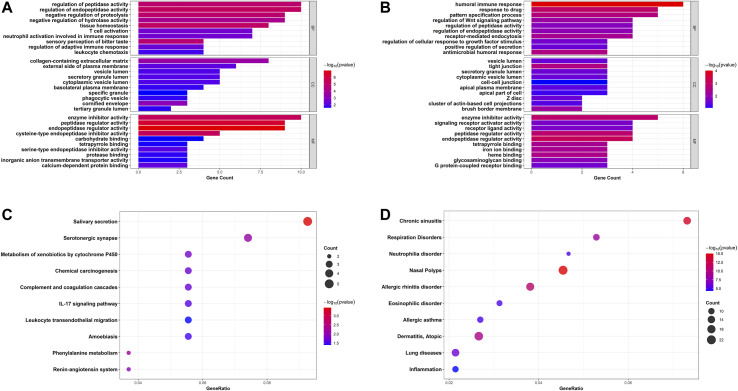
Bar plots and bubble charts of functional annotations involved in asthma. GO enrichment annotations of upregulated DEGs **(A)** and downregulated DEGs **(B)** in three categories: BP, CC, and MF; **(C)** KEGG pathway enrichment analysis of all DEGs; **(D)** Enrichment analysis of all DEGs in DisGeNET database. GO, Gene Ontology; BP, biological process; CC, cellular component; MF, molecular function; KEGG, the Kyoto Encyclopedia of Genes and Genomes.

GO annotation and KEGG pathway enrichment analyses of robust DEGs were performed to explore the biological classifications in asthma utilizing the clusterProfiler R package (Figures 4A–C). Three categories including biological process, cellular component, and molecular function were analyzed within up- and down-regulated DEGs, respectively. For 83 upregulated genes, the outcomes showed that changes in the biological process were mainly enriched in regulation of endopeptidase and peptidase activities, followed by response to tissue homeostasis, negative regulation of proteolysis. The significantly enriched entries for the cellular component part were collagen-containing extracellular matrix, cornified envelope, and external side of plasma membrane. Furthermore, endopeptidase and peptidase regulator activities, enzyme inhibitor activity, and cysteine-type endopeptidase inhibitor activity accounted for the majority of the molecular function terms (Figure 4A). In terms of 44 downregulated genes, the significantly enriched biological process terms were humoral immune response, response to drug, and pattern specification process. In the cellular component part, the downregulated genes were particularly enriched in tight junction, brush border membrane, and Z disc. Meanwhile, endopeptidase and peptidase regulator activities, enzyme inhibitor activity, and heme binding were mainly enriched in the molecular function group (Figure 4B). In addition, integrated DEGs were mainly involved in salivary secretion, metabolism of xenobiotics by cytochrome P450, IL-17 signaling pathway, and leukocyte transendothelial migration in KEGG pathway analysis (Figure 4C). The DisGeNET database was further used to identify DEGs related diseases. As shown in Figure 4D, the result indicated that robust DEGs participated in the progression of various diseases, such as Nasal Polyps, Allergic rhinitis disorder, Allergic asthma, and Atopic Dermatitis, which were all related to allergic reactions and chronic inflammation (Figure 4D). Taken together, the above results indicated that the robust DEGs were mostly associated with asthma-related functions.

### Protein-Protein Interaction Network Construction, Clusters Analysis, and Hub Gene Identification

**FIGURE 5:**
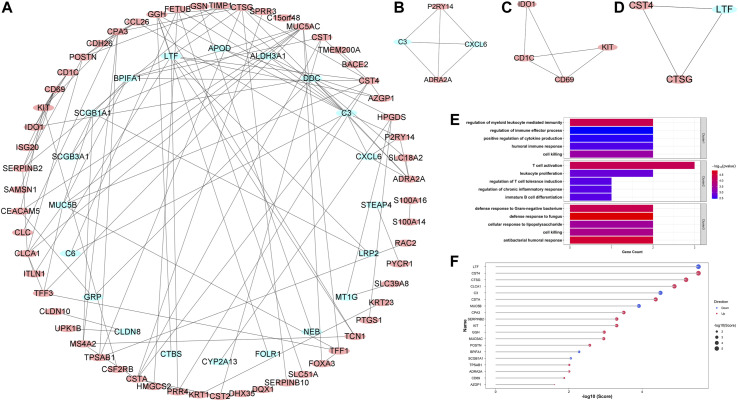
Protein-protein interaction (PPI) network construction, key clusters analyses, and hub genes identification. **(A)** The whole PPI network with all robust DEGs; **(B)** PPI network of Cluster 1; **(C)** PPI network of Cluster 2; **(D)** PPI network of Cluster 3. Red circles represented upregulated DEGs, while blue circles represented downregulated DEGs. **(E)** The significantly enriched entries for the biological process of three clusters; **(F)** The lollipop chart showed all hub genes identified by the RRA method. The red and blue dots represented the up- and down-regulated hub genes, respectively. Bigger dots represented higher ranks.

In order to explore the potential protein-protein interactions in asthma, all 127 robust DEGs were uploaded to the STRING database for further analysis (http://string.embl.de/). After hiding the disconnected nodes, the Cytoscape software was adopted to visualize the network (Figure 5A). As shown in the final network, 77 nodes and 114 edges were obtained, including 57 upregulated and 20 downregulated genes. Three key clusters were identified from the whole network using the MCODE plugin (Figures 5B–D). GO enrichment analyses showed that the significantly enriched biological process terms of three clusters were regulation of myeloid leukocyte mediated immunity, T cell activation, and antibacterial humoral response, respectively (Figure 5E).

**FIGURE 6:**
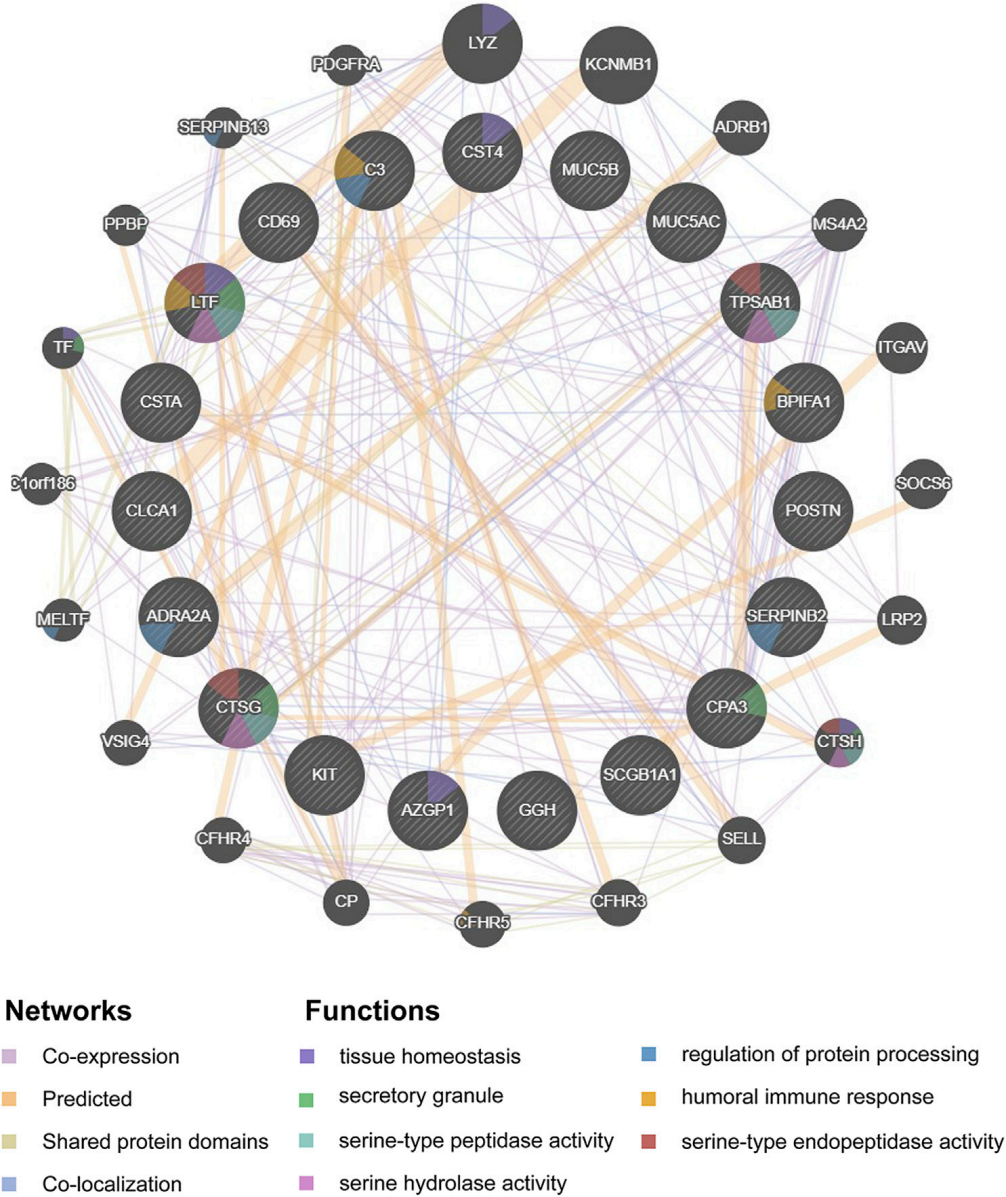
Protein-protein interaction network of the 19 hub-genes target network. Protein-protein interaction network of the 19 hub-genes target network was constructed using GeneMANIA database. The colors of the edges in the network indicated different bioinformatics methods used, including co-expression, website prediction, shared protein domains, and co-localization. The colors of the nodes in the network indicated the functional enrichment analysis of the query gene list.

Hub genes were subsequently screened out utilizing the cytoHubba plugin, which investigates the most important nodes in the PPI network with several topological analysis algorithms. In order to improve the positive rate of hub gene identification, the RRA method was used to integrate the top 50 ranked genes calculated by ten different topological analysis algorithms (MCC, DMNC, MNC, Degree, EPC, BottleNeck, EcCentricity, Closeness, Radiality, and Betweenness) and a total of 19 genes were obtained accordingly (Figure 5F). The detailed information of all 19 genes was listed in Table 2, including full names, scores of the RRA method, direction, and primary functions. The upset diagram of the top 50 ranked genes from the ten algorithms was shown in Supplementary Figure 1. The GeneMANIA database was used to construct the regulatory network among these 19 hub genes with functionally similar genes and the result showed that these genes may be involved in the following functions: tissue homeostasis, secretory granule, serine-type peptidase and endopeptidase activities, serine hydrolase activity, regulation of protein processing, and humoral immune response (Figure 6).

**TABLE 2:** The detailed information of 19 hub genes.

Symbol	Full name	Score	Direction	Primary function
CST4	Cystatin S	2.89E-06	Up	Antibacterial and antiviral activity
LTF	Lactotransferrin	2.89E-06	Down	Antimicrobial, antiviral, antifungal and antiparasitic activity
CTSG	Cathepsin G	6.31E-06	Up	Killing and digestion of engulfed pathogens
CLCA1	Chloride channel accessory 1	1.31E-05	Up	Mediating calcium-activated chloride conductance
C3	Complement C3	3.15E-05	Down	Modulating inflammation and possessing antimicrobial activity
CSTA	Cystatin A	4.25E-05	Up	Epidermal development and maintenance
MUC5B	Mucin-5 subtype B	1.22E-04	Down	Lubricating and viscoelastic properties of saliva and mucus
CPA3	Carboxypeptidase A3	3.16E-04	Up	Generating a mature protease released by mast cells
KIT	KIT proto-oncogene, receptor tyrosine kinase	4.94E-04	Up	Mediating proliferation, differentiation, migration, apoptosis and mast cell development
SERPINB2	Serpin family B member 2	4.94E-04	Up	Urokinase-type plasminogen activator inhibition
GGH	Gamma-glutamyl hydrolase	1.08E-03	Up	Hydrolyzing the polyglutamate sidechains of pteroylpolyglutamates
MUC5AC	Mucin-5 subtype AC	1.11E-03	Up	Protecting the mucosa from infection and chemical damage
POSTN	Periostin	2.68E-03	Up	Supporting adhesion and migration of epithelial cells
BPIFA1	BPI fold containing family A member 1	5.26E-03	Down	Antibacterial activity against Gram-negative bacteria
SCGB1A1	Secretoglobin family 1A member 1	8.86E-03	Down	Anti-inflammation, inhibition of phospholipase A2 and the sequestering of hydrophobic ligands
ADRA2A	Adrenoceptor alpha 2A	9.77E-03	Up	Adenylate cyclase inhibition
TPSAB1	Tryptase alpha/beta 1	9.77E-03	Up	Major neutral protease in mast cells
CD69	CD69 molecule	1.34E-02	Up	Lymphocyte proliferation
AZGP1	Alpha-2-glycoprotein 1, Zinc-binding	2.46E-02	Up	Antigen and peptide antigen binding

### circRNA-miRNA-mRNA Network Construction

**FIGURE 7:**
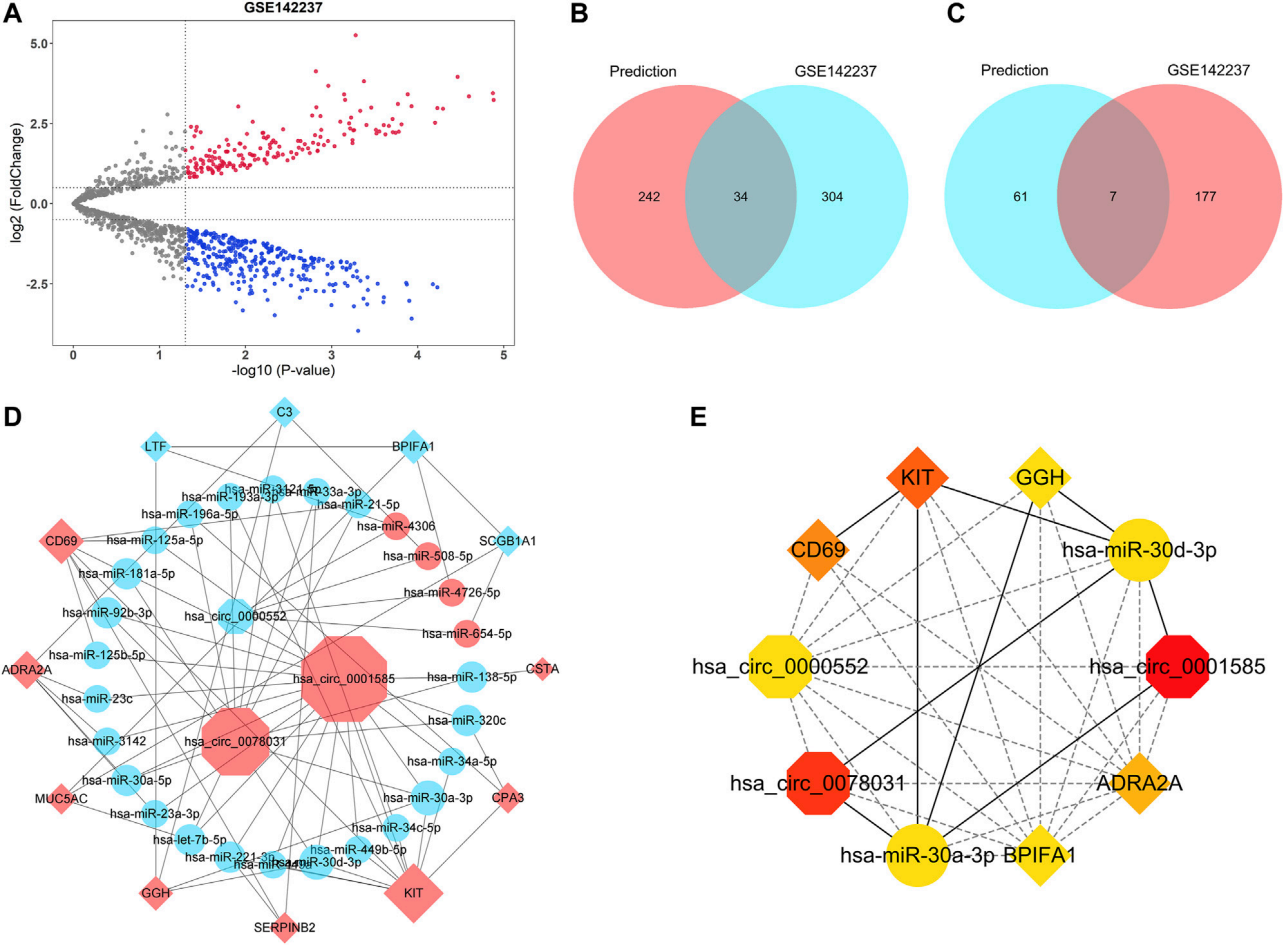
CircRNA-miRNA-mRNA network construction. **(A)** The volcano plot of differentially expressed miRNAs of GSE142237. The upregulated miRNAs were marked in red, while the downregulated miRNAs were marked in blue. The gray dots represented miRNAs with no significant difference. **(B)** The Venn diagram showed the intersection between the miRNAs targeting upregulated hub genes in the prediction result (red) and the downregulated miRNAs of GSE142237 (blue). **(C)** The Venn diagram showed the intersection between the miRNAs targeting downregulated hub genes in the prediction result (blue) and the upregulated miRNAs of GSE142237 (red). **(D)** The circRNA-miRNA-mRNA network. CircRNAs were marked as octagons, miRNAs were marked as ellipses, and mRNAs were marked as diamonds. The size of the nodes indicated the degree of the connection. Up-regulated molecules were marked in red, while down-regulated molecules were marked in blue. **(E)** The top 10 ranked ceRNAs identified by MCC algorithms. CircRNAs, miRNAs, and mRNAs were marked as octagons, ellipses, and diamonds, respectively. The colors of nodes represented the degree of the connection.

All 19 hub genes, including 14 upregulated genes (*CST4*, *CTSG*, *CLCA1*, *CSTA*, *CPA3*, *KIT*, *SERPINB2*, *GGH*, *MUC5AC*, *POSTN*, *ADRA2A*, *TPSAB1*, *CD69*, and *AZGP1*) and five downregulated genes (*LTF*, *C3*, *MUC5B*, *BPIFA1*, and *SCGB1A1*), were used for the circRNA-miRNA-mRNA network construction. MiRNA-mRNA analyses were performed with Targetscan, miRDB, and miRWalk databases. The intersection of miRNA results predicted by three databases was selected as the prediction result. Furthermore, GSE142237 was adopted to validate the prediction result. The volcano plot showed the distribution of DEMis of GSE142237 and there were 522 DEMis (184 upregulated and 338 downregulated) in asthma (Figure 7A). Generally, miRNA has a negative regulatory relationship with its targeted mRNA (Yekta et al., 2004; Lewis et al., 2005; Gebert and MacRae, 2019; Goodall and Wickramasinghe, 2021). Therefore, the miRNAs targeting upregulated hub genes further intersected with downregulated DEMis of GSE142237, while the miRNAs targeting downregulated hub genes intersected with upregulated DEMis. As shown in the Venn diagrams in Figures 7B,C and Table 3, there were 45 miRNA-mRNA relationships in total. The KEGG analyses of seven upregulated and 34 downregulated miRNAs were further conducted via the miRPathDB database (Supplementary Figure 2).

**TABLE 3:** 45 miRNA-mRNA relationships.

mRNA	mRNA direction	miRNA targeting mRNA
CST4	Up	hsa-miR-1293
LTF	Down	hsa-miR-4306
CTSG	Up	hsa-miR-3664-5p
C3	Down	hsa-miR-4472/hsa-miR-4447/hsa-miR-508-5p/hsa-miR-1275
CSTA	Up	hsa-miR-138-5p
CPA3	Up	hsa-miR-196a-5p/hsa-miR-4502/hsa-miR-320c
KIT	Up	hsa-miR-19a-5p/hsa-miR-34a-5p/hsa-miR-4699-3p/hsa-miR-148b-3p/hsa-miR-30a-3p/hsa-miR-148a-3p/hsa-miR-193a-3p/hsa-miR-34c-5p/hsa-miR-3121-5p/hsa-miR-449b-5p/hsa-miR-30d-3p/hsa-miR-449a/hsa-miR-4789-3p/hsa-miR-548v/hsa-miR-221-3p
SERPINB2	Up	hsa-miR-33a-3p/hsa-miR-221-3p
GGH	Up	hsa-miR-30a-3p/hsa-miR-30d-3p
MUC5AC	Up	hsa-let-7b-5p
BPIFA1	Down	hsa-miR-4726-5p/hsa-miR-1275
SCGB1A1	Down	hsa-miR-654-5p
ADRA2A	Up	hsa-miR-23a-3p/hsa-miR-30a-5p/hsa-miR-3142/hsa-miR-23c/hsa-miR-339-5p
CD69	Up	hsa-miR-125b-5p/hsa-miR-92b-3p/hsa-miR-181a-5p/hsa-miR-125a-5p/hsa-miR-21-5p/hsa-miR-4699-5p

Previous reports have demonstrated that circRNA could function as miRNA sponge to prevent co-expressed mRNA from miRNA-mediated degradation (Piwecka et al., 2017; Yu et al., 2017; Kleaveland et al., 2018; Kristensen et al., 2018; Wang et al., 2019a; Wong et al., 2020). Therefore, the target mRNA has the same expression pattern as circRNA. Consequently, 45 miRNA-mRNA relationships were used to construct the potential circRNA-miRNA-mRNA network. The corresponding circRNAs were predicted with the ENCORI database, while *hsa-miR-1293-CST4* and *hsa-miR-3664-5p-CTSG* failed to obtain targeted circRNAs. After cross-linking, only circRNAs that were able to regulate all remaining mRNAs by targeting corresponding miRNAs were retained to build the network. As was shown in Figure 7D, a ceRNA network, with three circRNAs, 27 miRNAs, and 12 mRNAs, was finally constructed. The detailed information of three circRNAs was listed in Supplementary Table 1. The top 10 ranked ceRNAs identified by MCC algorithm were shown in Figure 7E, including *hsa_circ_0001585*, *hsa_circ_0078031*, *hsa_circ_0000552*, *hsa-miR-30a-3p*, *hsa-miR-30d-3p*, *KIT*, *CD69*, *ADRA2A*, *BPIFA1*, and *GGH*.

## Discussion

In the present study, five microarray datasets from the GEO database were downloaded and analyzed by the RRA method to identify robust DEGs in primary epithelial cells between asthma patients and healthy controls. The RRA method is a both computationally efficient and statistically stable algorithm to combine multiple lists of genes from several datasets, which has four key features: robustness to noise, ability to handle incomplete rankings, assigning a significant score to each element in the resulting ranking, and efficient to compute (Kolde et al., 2012). However, the RRA method has not been reported to be used in asthma research yet. Using the RRA method, our study systematically integrated multiple microarray datasets on asthma in GEO. Furthermore, GO and KEGG enrichment analyses were conducted to annotate the functions of robust DEGs. In addition, the PPI network was constructed and key clusters were selected. In order to identify hub genes, the RRA method was utilized again to integrate the results of ten cytohubba plugin algorithms and nineteen genes were obtained. Potential miRNA-mRNA pairs were predicted by three miRNA databases (Targetscan, miRDB, and miRWalk) and further validated by a miRNA microarray dataset (GSE142237) to increase the reliability. By using the ENCORI database, a circRNA-miRNA-mRNA regulatory network was finally constructed.

The final ceRNA network included three circRNAs, 27 miRNAs, and 12 mRNAs. *KIT*, *CD69*, *ADRA2A*, *BPIFA1*, and *GGH* were subsequently identified as hub genes using the MCC algorithm. Of note, *BPIFA1* was among the top 10 ranked genes, while *KIT*, *CD69*, *ADRA2A*, and *GGH* ranked the 18th, the 20th, the 28th, and the 64th, respectively. Stem cell factor and its receptor, the *KIT* proto-oncogene receptor tyrosine kinase (henceforth known as *KIT*), is involved in mast cell development, migration, and function (Silva et al., 2006). Finotto and others found that the ligand of *KIT*, stem cell factor (*SCF*), played a critical role in a murine asthma model. Suppressing *SCF* expression in epithelial cells decreased various signs of lung inflammation (Finotto et al., 2001). In this study, *KIT* was also found to be significantly upregulated in bronchial epithelial cells. *CD69* is a type II transmembrane receptor, an activation marker of eosinophils. Kwon et al. reported that oleoylethanolamide increased *CD69* expression on purified eosinophils, thus playing a role in the pathogenesis of asthma by inducing eosinophilic airway inflammation (Kwon et al., 2021). Adrenoceptor Alpha 2A (*ADRA2A*) mediates the catecholamine-induced inhibition of adenylate cyclase through the action of G proteins. Yoshie et al. found that alpha-2 adrenoceptors existed in human airways and the overfunction of these receptors could cause intractable asthma (Yoshie et al., 1988). Bacterial permeability family member A1 (*BPIFA1*) is abundantly expressed in normal airway surface liquid and involved in the anti-inflammatory response. Thaikoottathil et al. found that *BPIFA1* inhibited airway eosinophilic inflammation by reducing eotaxin-2 production in alveolar macrophages (Thaikoottathil et al., 2012), which was consistent with Schaefer’s research (Schaefer et al., 2019). γ-glutamyl-hydrolase (*GGH*) is a ubiquitously expressed enzyme that regulates cell proliferation, DNA synthesis, and repair. However, the relationship between *GGH* and asthma has not yet been characterized, which requires further investigation. Numerous studies have concentrated on the diagnostic functions and therapeutic targets of these regulatory molecules for patients with asthma. Cahill et al. reported that both airway hyperresponsiveness and mast cell counts were decreased in patients with severe asthma after treated with imatinib, a *KIT* inhibitor (Cahill et al., 2017). It was also reported that anti-*CD96* mAb treatment could inhibit established airway inflammation as effectively as dexamethasone pretreatment in a mouse model of asthma (Wang et al., 2015). Sakai et al. found that the antagonist of *ADRA2A* might participate in the inhibition of the allergen provoked late asthmatic response (Sakai et al., 1995). However, there were no reports, so far, on the roles of *BPIFA1*, *GGH*, *hsa-miR-30a-3p*, *hsa-miR-30d-3p*, *hsa_circ_0001585*, *hsa_circ_0078031*, and *hsa_circ_0000552* in asthma.

Extensive studies have revealed that miRNAs expressed in bronchial epithelial cells contribute to asthmatic pathogenesis. In this study, *hsa-miR-30d-3p* and *hsa-miR-30a-3p* were identified in the final top 10 ceRNAs. Previous bioinformatic analyses showed that *hsa-miR-30d-3p* was associated with non-small cell lung cancer and inhibited epidermal growth factor receptor-targeted medicine therapy (Wang et al., 2017; Pan et al., 2019). *Hsa-miR-30d-3p* has also been implicated as a novel biomarker for treatment monitoring of postmenopausal osteoporosis (Weigl et al., 2021) and cerebral ischemia-reperfusion injury (Jin et al., 2021). However, *hsa-miR-30d-3p* has not been reported in asthma yet. *Hsa-miR-30a-3p* was reported to regulate the tumorigenesis in various cancer, such as gastric cancer (Wang et al., 2019b), lung adenocarcinoma (Wang et al., 2020), and pancreatic ductal adenocarcinoma (Shimomura et al., 2020). Li and others reported that *hsa-miR-30a-3p* regulates eosinophil activity through targeting CCR3 in asthma (Li et al., 2020).

*Hsa_circ_0001585*, *hsa_circ_0078031*, and *hsa_circ_0000552* were the three circRNAs finally identified in the ceRNA network. So far, there were no reports on these circRNAs. As shown in Supplementary Table 1, these circRNAs were mostly intergenic circRNAs with relatively long spliced lengths, bringing obstacles for research. However, the exceeding spliced length of these circRNAs could provide numerous miRNA response elements for miRNA to bind.

In summary, we included six microarray datasets (five mRNA datasets and one miRNA dataset) and utilized the RRA method to obtain robust DEGs and robust hub genes. Based on the prediction of three miRNA-related databases (Targetscan, miRDB, and miRWalk) and one circRNA-related database (ENCORI), an epithelial circRNA-miRNA-mRNA network was finally constructed and the top 10 epithelial ceRNAs were identified. This epithelial ceRNA network provides new clues for future study on airway epithelial cells in asthma.

## Data Availability

Publicly available datasets were analyzed in this study. This data can be found here: GEO database (https://www.ncbi.nlm.nih.gov/geo/).
