# Supplementary material for: A Potential circRNA-miRNA-mRNA Regulatory Network in Asthmatic Airway Epithelial Cells Identified by Integrated Analysis of Microarray Datasets
Source: Front Mol Biosci. 2021 Jul 16;8:703307. doi: 10.3389/fmolb.2021.703307 (PMC8322703; doi:10.3389/fmolb.2021.703307)
Supplement: Supplementary file 1 [file DataSheet1.docx]

**Supplementary Material**

Supplementary Table 1

| Circbase ID | Gene | Chr | Start | End | Strand | Spliced Length |
| --- | --- | --- | --- | --- | --- | --- |
| hsa_circ_0001585 | HIST1H3D | 6 | 26124709 | 26217451 | - | 91153 |
| hsa_circ_0078031 | - | 6 | 141940243 | 142231148 | - | 290905 |
| hsa_circ_0000552 | - | 14 | 71880664 | 71948928 | + | 68264 |

Supplementary Figure 1


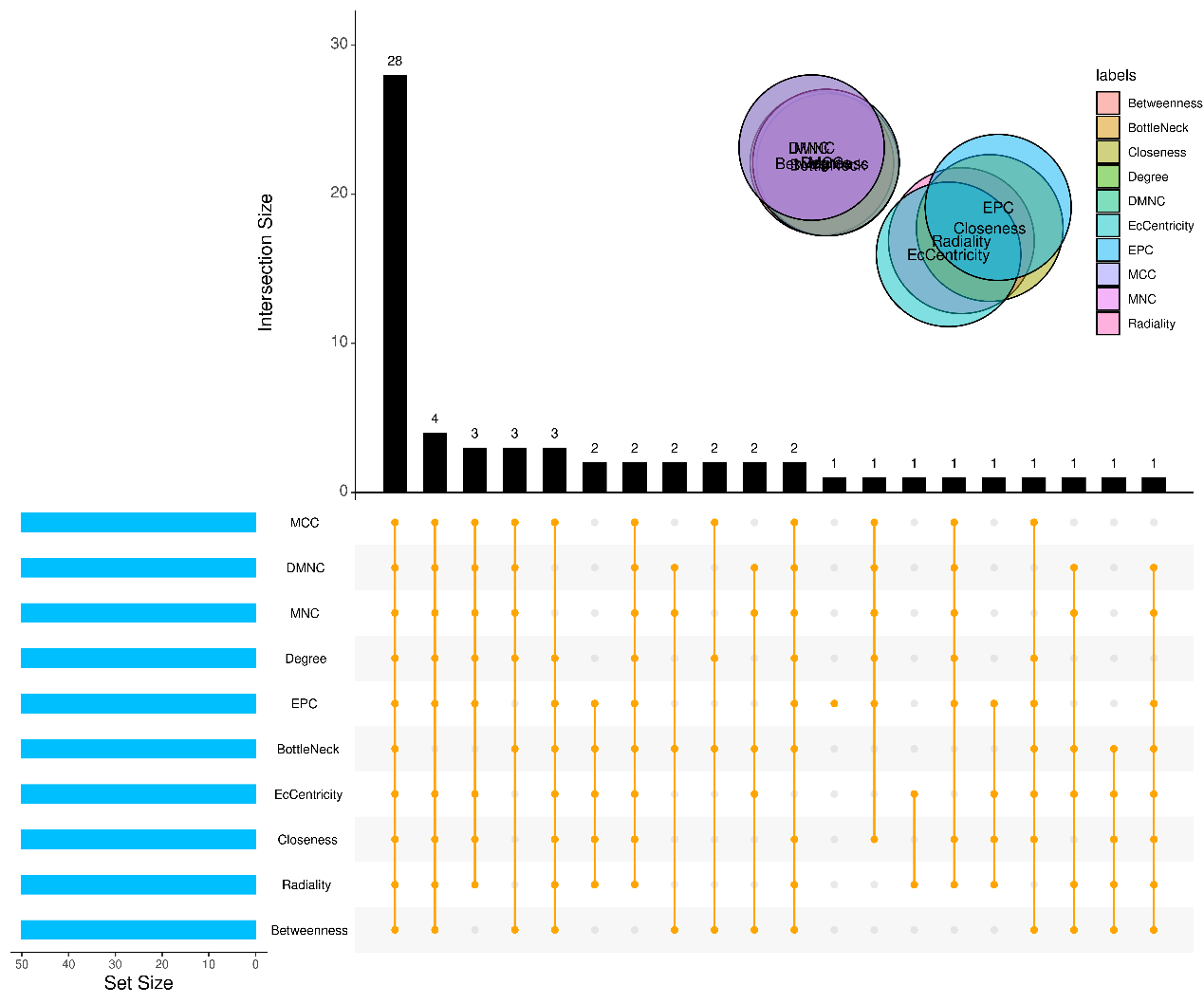


Supplementary Figure 1. The upset and Venn diagrams showed the top 50 ranked genes calculated by ten algorithms, including Maximal Clique Centrality (MCC), Density of Maximum Neighborhood Component (DMNC), Maximum Neighborhood Component (MNC), Degree, Edge Percolated Component (EPC), BottleNeck, EcCentricity, Closeness, Radiality, and Betweenness.

Supplementary Figure 2


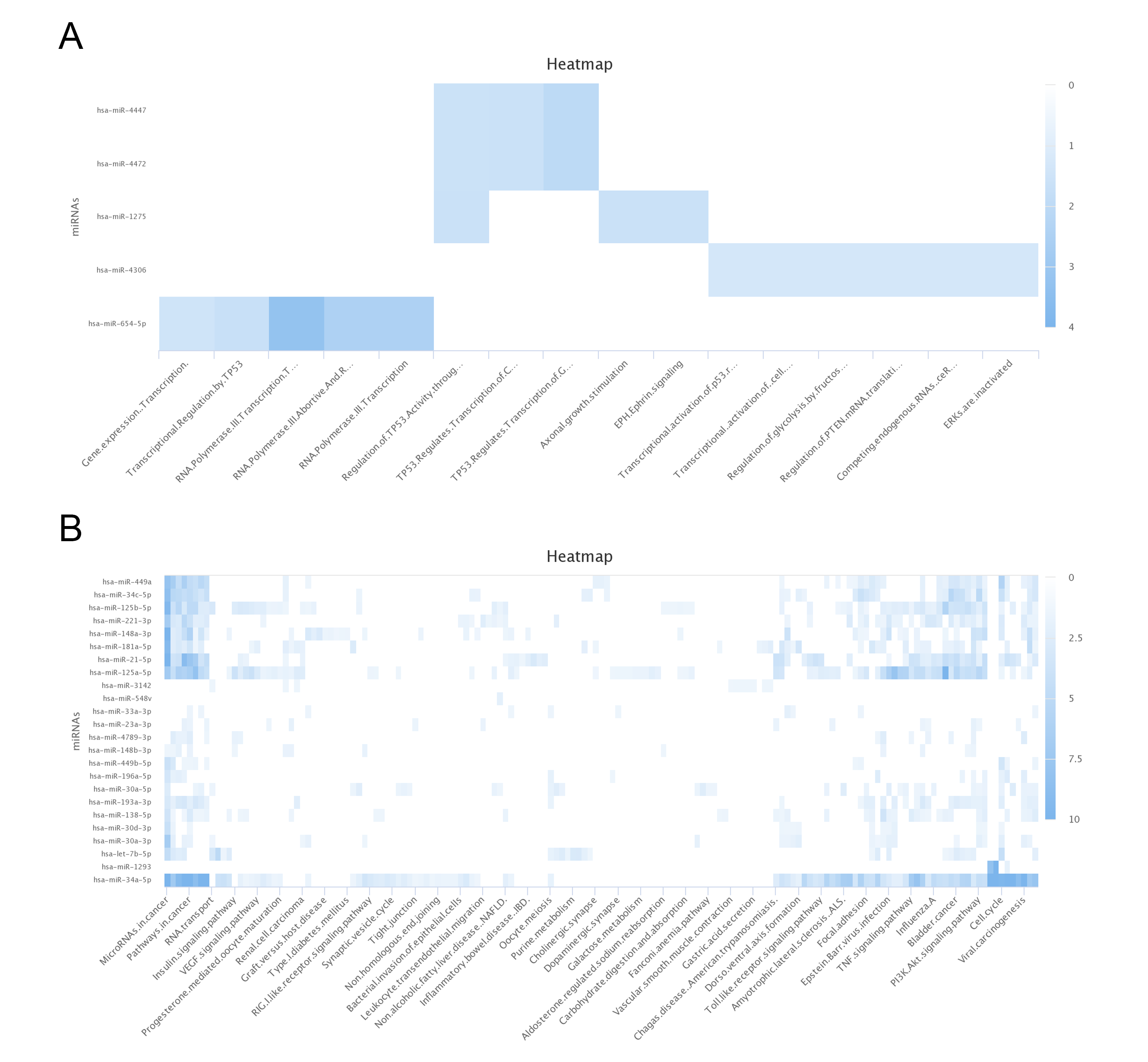


Supplementary Figure 2. The heatmap visualization depicted the enrichment results of (A) 7 upregulated and (B) 34 downregulated miRNAs involved in the miRNA-mRNA relationships for the category of the KEGG database. Rows represented the enrichment results for the targets. Columns represented all KEGG pathways significant for different miRNAs. The color of each field represented the -log10 transformed *P*-value and darker colors indicated more significant associations between miRNA and the corresponding pathway.
